# Supplementary material for: Leishmania naiffi and lainsoni in French Guiana: Clinical features and phylogenetic variability
Source: PLoS Negl Trop Dis. 2020 Aug 14;14(8):e0008380. doi: 10.1371/journal.pntd.0008380 (PMC7449503; doi:10.1371/journal.pntd.0008380)
Supplement: S1 Table — (DOCX) [file pntd.0008380.s002.docx]

| Patient ID | 1 | 2 | 3 | 4 | 5 |
| --- | --- | --- | --- | --- | --- |
| Gender | M | M | M | M | M |
| Place of Birth | French mainland | French mainland | French Caribbean | French Guiana | French mainland |
| Age at diagnosis | 38 | 57 | 39 | 53 | 39 |
| Occupation | Teacher | Military | Building and public work | Office worker | Deliverer |
| Site of probable contamination | Coastal region  (St Laurent du Maroni) | Coastal region  (Primary forest) | Coastal region  (Cayenne) | Coastal region  (Regina) | Coastal region  (Remire Montjoly) |
| Probable month of contamination | January | March | May | August | April |
| Time-to-diagnosis, months | 2 | 1 | 59 | 2 | 3 |
| Number of lesions, (n) | 1 | 1 | 1 | 1 | 1 |
| Semiology | Ulcer | Ulcer | Nodule | Ulcer | Ulcer |
| Localization | Upper limb | Lower limb | Upper limb | Upper limb | Lower limb |
| Adenopathy | 0 | 0 | 0 | 0 | 0 |
| Lymphangitis | 0 | 0 | 0 | 0 | 1 |
| First line treatment | Abstention | Pentamidine | Pentamidine | Abstention | Abstention |
| Outcome | Cure | Cure | Failure | Cure | Cure |
| Second line treatment | None | None | Meglumine antimoniate | None | None |
| Outcome | - | - | Cure | - | - |
