# Supplementary material for: Leishmania naiffi and lainsoni in French Guiana: Clinical features and phylogenetic variability
Source: PLoS Negl Trop Dis. 2020 Aug 14;14(8):e0008380. doi: 10.1371/journal.pntd.0008380 (PMC7449503; doi:10.1371/journal.pntd.0008380)
Supplement: S2 Table — (DOCX) [file pntd.0008380.s003.docx]

| Patient ID | 1 | 2 | 3 | 4 | 5 | 6 | 7 | 8 | 9 | 10 | 11 | 12 | 13 |
| --- | --- | --- | --- | --- | --- | --- | --- | --- | --- | --- | --- | --- | --- |
| Gender | M | M | F | F | F | M | F | M | F | M | M | F | M |
| Place of Birth | French Guiana | French Guiana | Brazil | Algeria | Brazil | Brazil | Brazil | Brazil | Brazil | Brazil | Brazil | Brazil | French mainland |
| Age at diagnosis | 3 months | 1 | 32 | 53 | 29 | 63 | 41 | 35 | 48 | 16 | 36 | 24 | 29 |
| Occupation | None | None | NS* | Teacher | None | Forester | NS | Gold miner | Works on a gold camp | NS | NS | NS | Doctor |
| Site of probable contamination | Maroni region | Center region | Maroni region | Maroni region | Maroni region | Oyapock  region | Maroni region | Maroni region | Maroni region | Center region | Maroni region | Maroni region | Coastal region |
| Probable month of contamination | February | November | July | July | NS | December | NS | May | February | NS | NS | NS | January |
| Time-to-diagnosis, months | 3 | 1 | 1 | 3 | NS | 3 | NS | 1,5 | 1 | NS | NS | NS | 1 |
| Number of lesions, (n) | 1 | 3 | 1 | 1 | 1 | 1 | 1 | 3 | 1 | NS | 1 | 2 | 2 |
| Semiology | Ulcer | Ulcer | NS | Ulcer | Nodule | Ulcer | NS | Ulcer | Ulcer | NS | Ulcer | Ulcer | Ulcer |
| Localization | Lower limb | Upper and lower limbs | Lower limb | Upper limb | Upper limb | Upper limb | Upper limb | Upper and lower  limbs | Lower limb | NS | Lower limb | Upper limb | Neck |
| Adenopathy | 0 | 0 | NS | 0 | NS | 0 | NS | NS | 1 | NS | 0 | 0 | 0 |
| Lymphangitis | 0 | 0 | NS | 0 | NS | 0 | NS | NS | 1 | NS | 0 | 0 | 0 |
| First line treatment | Abstention | Pentamidine | NS | Pentamidine | NS | NS | NS | NS | Pentamidine | NS | Pentamidine | Abstention | Pentamidine |
| Outcome | Cure | Cure | NS | Failure | NS | NS | NS | NS | NS | NS | Cure | NS | Cure |
| Second line treatment | None | None | - | Meglumine antimoniate | - | NS | NS | S | NS | NS | None | NS | None |
| Outcome | - | - | - | Cure | - | - | - | - | - | - | - | - | - |

| Patient ID | 14 | 15 | 16 | 17 | 18 | 19 | 20 | 21 | 22 | 23 | 24 | 25 |
| --- | --- | --- | --- | --- | --- | --- | --- | --- | --- | --- | --- | --- |
| Gender | M | M | F | M | M | F | F | M | F | M | F | F |
| Place of Birth | French Guiana | NS | Brazil | Brazil | Brazil | Brazil | French Guiana | Brazil | French mainland | Brazil | Brazil | Brazil |
| Age at diagnosis | 10 | 31 | 30 | 30 | 48 | 24 | 6 | 32 | 47 | 9 months | 38 | 41 |
| Occupation | None | NS | NS | Building and public work | Gold miner | NS | None | Gold miner | Gold miner | None | Works on a gold camp | NS |
| Site of probable contamination | Coastal  region | Maroni region | Maroni region | Oyapock  region | Center  region | Maroni  region | Coastal region | Center region | Coastal region | Maroni region | Maroni region | Maroni region |
| Probable month of contamination | June | June | NS | January | July | January | December | April | May | May | June | NS |
| Time-to-diagnosis, months | 3 | 0,5 | NS | 3 | 5 | 1 | 0,75 | 7 | 2 | 1 | 3 | NS |
| Number of lesion, (n) | 1 | >3 | 2 | 3 | 1 | 2 | 2 | 2 | 2 | 1 | 1 | 1 |
| Semiology | Ulcer | Ulcer | Ulcer | Ulcer | Ulcer | Ulcer | Ulcer | Ulcer | Ulcer | Ulcer | Nodule | Ulcer |
| Localization | Lower limb | Upper limb | Upper and lower limbs | Lower limb | Lower limb | Upper limb | Neck and Upper limb | Upper  limb | Upper limb | Head | Head | Lower limb |
| Adenopathy | 1 | 1 | NS | 1 | 1 | NS | 1 | 0 | 0 | 0 | 0 | 0 |
| Lymphangitis | 0 | 0 | NS | 0 | 0 | NS | 0 | 0 | 1 | 0 | 0 | 0 |
| First line treatment | Pentamidine | Pentamidine | Pentamidine | Meglumine antimoniate | Pentamidine | NS | Pentamidine | Pentamidine | Pentamidine | NS | Pentamidine | Pentamidine |
| Outcome | Cure | NS | Cure | Failure | Partial response | NS | Cure | Partial response | NS | NS | Partial response | Partial response |
| Second line treatment | None | NS | None | Pentamidine | Pentamidine | NS | None | Pentamidine | NS | NS | Pentamidine | Pentamidine |
| Outcome | - | - | - | Cure | Cure | - | - | Cure | - | - | NS | Cure |
