# Supplementary material for: Leishmania naiffi and lainsoni in French Guiana: Clinical features and phylogenetic variability
Source: PLoS Negl Trop Dis. 2020 Aug 14;14(8):e0008380. doi: 10.1371/journal.pntd.0008380 (PMC7449503; doi:10.1371/journal.pntd.0008380)
Supplement: Attachment — Submitted filename: Answer letter.docx [file pntd.0008380.s004.docx]

Cayenne, 28^th^ March 2020

To the Editor-in-Chief of *PLOS Neglected Tropical Diseases,*

Dear Editor,

Please find enclosed a revised version of our manuscript entitled “***Leishmania naiffi* and *lainsoni* in French Guiana: clinical features and phylogenetic variability**”, which we would like to submit for publication as Original Article in *PLOS Neglected Tropical Diseases*.

**Comments from the reviewers have been addressed as follows:**

Reviewer #1: This is a descriptive study showing several characteristics associated with cutaneous leishmaniasis caused by L. lainsoni and L. naiffi in French Guiana. Although the study is focusing in French Guiana, the similarity observed for strains of each species with strains from other localities indicates that the discussion presented is relevant for the understading of the disease caused by both parasites.

Reviewer #2: The methods are clearly described and adequate. The sample size is low but this is inherent to the study of a very rare disease; and actually, the cohort is of an interesting size for the Leishmania species studied.
It is written that "diagnosis" was performed using PCR-RFLP, hsp70 sequencing or MALDI-TOF. I understand that species typing was done using these methods, but not diagnosis. Could the authors specify how the initial/primary diagnosis was made (first arrow of Fig.1)?

- We modified this paragraph accordingly:

“Positive diagnosis was defined by positive smear or culture, or the presence of DNA amplification during PCR-RFLP or Hsp70 PCR. Species identification was then made with MALDI-TOF on positive cultures and/or species-specific bands on PCR-RFLP and/or Hsp70 sequencing.”

The in vitro cultivation method is not indicated.

- We added the following sentence: “Biopsies were cultivated in RPMI medium during up to 21 days at 28°C.”

This reviewer is not competent to assess the validity of the phylogenetic methods and inferences.

--------------------

**Results**
-Does the analysis presented match the analysis plan?
-Are the results clearly and completely presented?
-Are the figures (Tables, Images) of sufficient quality for clarity?

Reviewer #1: It is important to clarify the identification of L. naiffi parasites. It was mentioned that all L. naiffi cases resulted in positive cultures, but one failed for hsp70 PCR. Why? I can't see a reason for PCR hsp70 PCR fail when DNA from Leishmania culture is employed.
It is better to try to re-write the topic identification of Leishmania species as this is a bit confused to follow.

- We thank the reviewer for this comment and agree. We have re-written this paragraph.

What we meant was all cultures were initially positive at the time of diagnosis. However, due to logistical and technical issues in the hospital and the university, most of these cultures could not be retrieved at the time of study. Therefore, DNA samples which had been properly stored were used for the study. Some of these samples failed to provide satisfying amplification.

The fact that L. lainsoni was frequently more associated with patients born in Brazil is correctly associated with professional activity as gold mining. It is already known the association between Brazilian gold miner and CL in French Guiana. I suggest not to link L. lainsoni patients to the fact that they were born in Brazil, as what is significant here is the professional activity.

- We thank the reviewer for this comment and agree. We have rephrased our statements accordingly.

Why for L. lainsoni all the socio-demographic and clinical characteristics were presented in general in Table 1 and for L. naiffi a detailed table was presented for each patient? Table 2 should include information on L. lainsoni patients and presented as Supplementary Table.

- We thank the reviewer for this comment. Table 2 has been completed to include information on *L. lainsoni* patients and is now presented as Supplementary Table S1 and S2.

Phylogenetic analysis
Although it is large employed the term “phylogenetic analysis” for the analysis conducted in this study, my suggestion is to replace that for “HSP70 and cytb partial nucleotide sequence analysis”.  The idea here was not to infer about evolutionary relationship, but to perform a good explanatory analysis employing distance based method.

- We agree with the reviewer on this matter. The terms “*phylogenetic analysis*” have been replaced with “*Hsp70 and cytb partial nucleotide sequence analysis*” throughout the text.

I suggest to present the analysis performed with HSP70 and cytb genes in the same figure, as A and B. The results from cytb were not completely concordant to hsp70 and this must be considered to define the groups, although the sequences analyzed are not exactly the same. For example, cluster IV considered as a group for L. lainsoni using hp70 sequences is not supported by bootstrap values in the hsp70 tree or by cytb analysis.

- Analysis from both target genes are now presented in the same figure. Hsp70 was solely used for group definitions due to the less efficient DNA amplification with cyt b and the several amplification failures. However, due to the discordance with bootstrap values, we agreed to rephrase more cautiously our definition of the grouped cases previously defined as “Cluster IV”.

Reviewer #2: The Results are clearly and completely presented.
Although I understand that infections due to L. naiffi are particularly rare, the low number of cases due to L. naiffi is a problem for inferring any conclusions about differences between the two species. The sentence "L.  (V.)  lainsoni  was  more frequently responsible for multiple lesions (11 cases) than L. (V.) naiffi (0 case)" should be attenuated (e.g. "seems to be").

- We agree with the reviewer and have attenuated our sentences accordingly.

Statistical considerations made from 5 cases cannot be put forward. In that sense, the authors took care to not write that there were more paediatric infections due to L. naiffi than to L. lainsoni, but more cautiously, that 20% of infections due to L. lainsoni were observed in children. The same caution should be applied throughout the paper.

- Statistical considerations have been removed throughout the paper.

The probable month of contamination should be indicated whenever possible for all cases. This is easy to calculate from the date of diagnosis and the delay between onset of symptoms and diagnosis. It would allow some considerations about the period of contamination for both species.

- We agree with the reviewer and have indicated the probable month of contamination (Supplementary Table S1 and S2).

Spontaneous healing of the lesions is mentioned several times throughout the paper. It would be good if this was summarized for all cases with the clinical course addressed in page 11.

- We agree with the reviewer and have summarized the cases of spontaneous healing in Table 1.

This reviewer is not competent to assess the validity of the phylogenetic methods and inferences. However, given the very low genetic distance observed between both clusters (evidenced by the existence of a single base mutation), the isolation of cluster IV versus cluster III may be a bit artificial? This distinction should perhaps be justified in the text.
-	We completely agree that this distinction might seem a bit artificial. However, clinical/geographical distinctions between grouped cases of cutaneous leishmaniasis can be linked to a very small number of mutations. We think it remains noteworthy to compare these two groups of strains but agreed to attenuate our statements and acknowledge that the definition of cluster might not be entirely fulfilled.

--------------------

**Conclusions**

-Are the conclusions supported by the data presented?
-Are the limitations of analysis clearly described?
-Do the authors discuss how these data can be helpful to advance our understanding of the topic under study?
-Is public health relevance addressed?

Reviewer #1: The authors should try to re-write this part. Some statements seem not adequate to be presented here. For example, this study does not allow concluding that “L. lainsoni seems to infect children more frequently than other species”. There is also some results presented as conclusion, like “A mild clinical course was observed for all cases of L. (V.) naiffi”. The second paragraph must be completely revised.

-	The Conclusion has been completely revised and reduced.

Reviewer #2: The conclusions of the study are well supported by the data presented. The limitations of the sudy are also well described. The Discussion provide an extensive literature search.
However, the Discussion is lengthy and would benefit from being shortened. For example, the considerations about the immune response in children (page 21) may be deleted. Also the Discussion rewrites several points of the Results, which appears redundant.

-	These considerations have been deleted, along with several redundant points.

The Conclusion is redundant and may be deleted (except the sentence about "determination   of   vector–parasite–reservoir relationships "). The last sentence of the conclusion is useless (We showed  the identification of different genotypes within L. (V.) naiffi and L. (V.) lainsoni and how these genotypes are distributed at the geographic level.).

- The Conclusion has been completely revised and reduced, as mentioned above.

  --------------------
- **Editorial and Data Presentation Modifications?**
  Use this section for editorial suggestions as well as relatively minor modifications of existing data that would enhance clarity. If the only modifications needed are minor and/or editorial, you may wish to recommend “Minor Revision” or “Accept”.

  Reviewer #1: English / typing revision is required.
- A thorough revision by an English native speaker has been performed.

Reviewer #2: Abstract : "percentage is missing in "A high  number  of  pediatric  cases  (n=5;  %)  was  observed ".
Page 10: the sentence "Patients  infected  by  L.  (V.)  lainsoni were  significantly  more frequently born in Brazil" is perturbing as it is placed in the middle of a paragraph exposing the whole of the data without considering the species.
Page 10-11: the regions mentioned in the text should refer to Figure 3 for the reader who does not have a good knowledge of French Guiana: it would help to a better understanding of the geographical origin of the cases mentioned here.

- The regions mentioned in the text now refer to Figure 3.

Discussion : first paragraph: remove "comprehensive", just write "new data".
A few minor English errors in the Abstract , Introduction, Fig. 1,  Results etc. (have been/were, was/has been, consultation, theses, register, a  second  therapeutic  line  by  a  new  injection…).

- All these mistakes have been corrected. We thank the reviewers for pointing them out.

Note: the absence of line numbering makes it difficult (or time consuming) to address very minor points such as English errors.

- We apologize for this mistake. Line numbers have been added to the text.

  --------------------

  **Summary and General Comments**
  Use this section to provide overall comments, discuss strengths/weaknesses of the study, novelty, significance, general execution and scholarship. You may also include additional comments for the author, including concerns about dual publication, research ethics, or publication ethics. If requesting major revision, please articulate the new experiments that are needed.

  Reviewer #1: All comments were presented in the previous topics.

  Reviewer #2: This manuscript addresses the question of cutaneous leishmaniosis due to two rare species of Leishmania in French Guiana. Although, precisely due to the scarcity of such infections, the number of cases included in the study is limited, the authors provide new data about clinical and epidemiological features of CL due to L. (V.) naiffi and L. (V.) lainsoni in French Guiana. They also show novel phylogenetic data about the genetic diversity between and within both Leishmania species. As such, the manuscript is interesting and worth publishing.
  Yet, the authors should address a number of comments before publication (see other sections above).

We hope these modifications will meet the reviewers’ expectations. We thank you again for considering this manuscript.

Best regards,

Dr Romain Blaizot

Dermatology Department, Andrée Rosemon Hospital

Avenue des Flamboyants, 97300 CAYENNE

French Guiana

[Blaizot.romain@gmail.com](mailto:Blaizot.romain@gmail.com)

Tel : +594 594 39 53 59

Fax : +594 594 39 52 83
